# Modern Endoscopic Imaging in Diagnosis and Surveillance of Inflammatory Bowel Disease Patients

**DOI:** 10.1155/2018/5738068

**Published:** 2018-05-31

**Authors:** Gheorghe Hundorfean, Stephen P. Pereira, John G. Karstensen, Peter Vilmann, Adrian Saftoiu

**Affiliations:** ^1^Department of Medicine I, Ludwig Demling Endoscopy Center of Excellence, University of Erlangen-Nuremberg, Erlangen, Germany; ^2^Institute for Liver and Digestive Health, University College London, Royal Free Hospital Campus, London, UK; ^3^Department of Endoscopy, Gastrointestinal Unit, Copenhagen University Herlev Hospital, Herlev, Denmark; ^4^Research Center of Gastroenterology and Hepatology, University of Medicine and Pharmacy of Craiova, Craiova, Romania

## Abstract

Endoscopy remains the most important diagnostic and monitoring modality in the management of inflammatory bowel disease. Advances in imaging have progressively added new tools into the armamentarium of endoscopists with the goal of more accurate, sensitive, and accessible visual diagnoses for the benefit of patients with gastrointestinal diseases. Here, we review the relevant literature regarding commonly used endoscopic techniques (dye-based and digital chromoendoscopy, high-definition endoscopy, capsule endoscopy, and endosonography), as well as advanced and experimental technologies (full-spectrum endoscopy, endocytoscopy, autofluorescence, laser endoscopy, and endomicroscopy, including molecular imaging), applicable to inflammatory bowel diseases and emerging for implementation into everyday practice. Additionally, we discuss future directions and techniques as candidates for a superior inflammation imaging in the diagnosis and prediction of therapeutic response.

## 1. Introduction

Patients with inflammatory bowel disease (IBD), comprising ulcerative colitis (UC), and Crohn's disease (CD) need regular endoscopic evaluation for the assessment and monitoring of the extent and severity of inflammation, therapeutic response, and surveillance for colorectal carcinoma (CRC) [1]. Currently, a multitude of endoscopic imaging techniques fulfil these necessities based on guideline recommendations. In addition to standard techniques like high-definition white light endoscopy (HD-WLE), emerging experimental techniques like confocal laser endomicroscopy (CLE) and multiphoton imaging enrich the endoscopic toolbox providing vivid and high-resolution images in real time. Individual improvements in adenoma detection rates are pivotal for adoption and implementation of new techniques. In this regard, the awareness of interval cancer in IBD [2] must increase the efficiency of surveillance endoscopies in IBD patients and focus on higher proficiency in learning and implementing novel endoscopic technologies in the clinical routine.

The aim of this review is to highlight the current technologies available for conventional as well as advanced endoscopic imaging, both for assessment of inflammation and for neoplasia detection in IBD.

## 2. Current Recommendations

Screening colonoscopy and therapeutic polypectomy significantly reduce the risk of colorectal carcinoma [3]. The widest endoscopic management strategy of CRC screening in IBD relied initially on the random biopsy technique published by the American Gastroenterology Association in 2010 [4]. A 2013 European guideline regarding IBD screening and surveillance management recommended targeted biopsies of macroscopically visible lesions and 2–4 random biopsies every 10 cm within the colon [5]. In 2015, an international consensus statement (SCENIC) recommended chromoendoscopy (CE) as the preferred endoscopic technique for dysplasia detection and surveillance [6] in IBD, which is the current standard. National society guidelines, like those of the British Society for Gastroenterology (BSG, 2010), also advocate dye pan-chromoendoscopy with targeted biopsy as the technique of choice and not virtual CE like NBI. If dye-CE is not available, the BSG suggests the old recommendation of 2–4 biopsies every 10 cm of the colon and rectum [7]. In the present high-definition and targeted technology era, the standard in endoscopic practice in referral centers shifted in the last years towards the virtual/digital chromoendoscopy. In this regard, if a chromoendoscopic evaluation (dye/virtual) with biopsy of the inflamed segments is not possible/not available, a targeted high-definition white light endoscopy is to be evaluated as a superior alternative to the random biopsies. If any of the chromoendoscopic techniques (dye or virtual) or high-definition endoscopy are not available, then the patient should be handled according to the BSG recommendation, as minimum standard. If multiple biopsies have to be avoided because of bleeding complication/anticoagulants and so on, a prodigious standard definition assessment remains as last option (with biopsies from any polypoid structure or suspect surface deformity or inhomogenicity other than classical pseudopolyps) in the case of very high confidence and experience. Alternatively, the patient should be referred to an endoscopic unit with an appropriate chromoendoscopy of HD technology for a second endoscopic evaluation.

## 3. Commonly Used Endoscopic Imaging Techniques

### 3.1. High-Definition White Light Endoscopy

**Figure 1:**
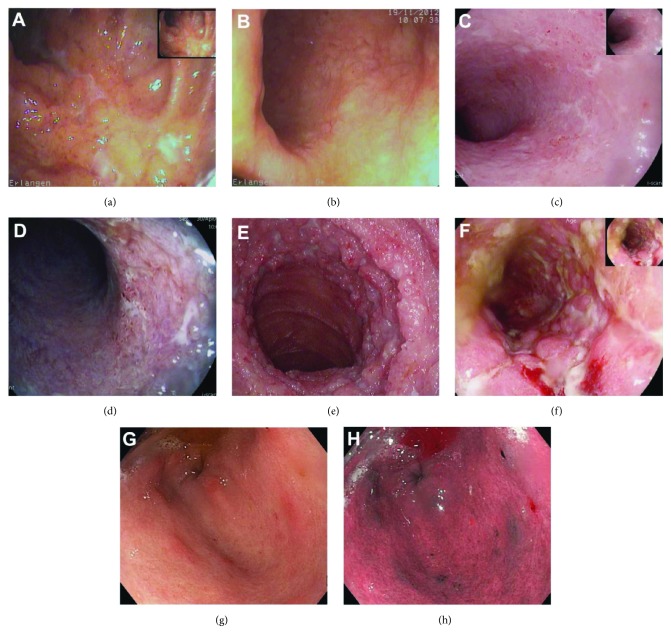
Endoscopic images depicting Crohn's disease (CD) and ulcerative colitis (UC) lesions with different techniques and resolutions: image (a) shows a moderate to severe Crohn's colitis, while (b) shows a mild UC (endoscopic Mayo 1), both in standard definition. Images (c) and (d) depict again a distal UC involvement in high-definition white light (HD-WLE) as well as in digital chromoendoscopy using *i*-Scan (Mayo 1–2). In comparison, pictures (e) and (f) show a severe CD of the ileum and colon (high-definition WLE and *i*-Scan). Images (g) and (h) show high-definition WLE and narrow-band imaging (NBI) (h) of a gastric CD, highlighting erosions and aphthoid lesions.

High-definition white light endoscopy (HD-WLE, Figures 1 and 1(e)–1(g)) was introduced in 1993 and is the current standard in gastrointestinal endoscopic practice that replaced standard-definition video endoscopy (Figures 1(a) and 1(b)). HD-WLE allows a resolution of more than 1 million pixels per image and can be visualized on a HD screen. HD-WLE increases the adenoma detection rate compared to standard definition [8] in the general population.

### 3.2. Chromoendoscopy (CE)

Chromoendoscopy (CE) is the most widespread imaging technique for IBD screening and is anchored in the latest international guideline (SCENIC) [6]. CE in the colon comprises dye-based chromoendoscopy (methylene blue and indigo carmine) and digital chromoendoscopy (optical and virtual CE).

#### 3.2.1. Dye-Based CE

Dye-based CE permits characterization of mucosal lesions by topical stain application. One of the first descriptive methylene blue-aided CE studies was done in ulcerative proctitis in 1979 by Baldi and co-workers [9]. A good correlation of UC inflammation severity in CE with topical methylene blue and indigo carmine compared to conventional histology was shown in 25 UC patients by Ibarra-Palomino et al. [10]. Beyond the standard white light endoscopy (WLE), studies using dye-based CE with magnification endoscopes (cresyl violet plus zooming) proved a lower clinical and histologic inflammation in UC patients having cryptal openings and a network pattern [11]. The first large study comparing methylene blue-based CE-targeted biopsies versus WLE with random biopsies was published by Kiesslich and co-workers proving a superiority of CE in neoplasia detection [12]—a conclusion later confirmed by other studies using indigo carmine CE [13]. Further prospective studies have underlined the superiority of CE in comparison to standard-definition endoscopy for adenoma detection both in IBD and in the general population [14–16].

#### 3.2.2. Dye-Less Digital Chromoendoscopy

Dye-less digital chromoendoscopy uses artificially staining techniques which add colour by pressing a button and subsequently enhancing the mucosal contrast. This is performed either through optical filters, so-called *optical chromoendoscopy*, like the narrow-band imaging technique (NBI, Olympus, Japan; Figure 1(h)) [17, 18] or by virtual video postprocessing in real time, so-called *virtual chromoendoscopy* (*i*-Scan, PENTAX, and FICE, Fujinon, Japan; Figure 1(d)) [19].

Concerning virtual chromoendoscopy, Hofmann and collaborators compared conventional HD-WLE with *i*-Scan and classical CE (with methylene blue) in neoplasia detection. In this setting, *i*-Scan imaging proved equal to methylene blue-aided CE in identifying neoplastic lesions [20]. Furthermore, neoplasia detection using colonoscopy with *i*-Scan was superior to standard colonoscopy [21]. *i*-Scan imaging in comparison to histology results also provided a more precise assessment of inflammation in IBD patients [22]. This fact allows a targeted bioptic sampling from pathological mucosal areas for disease confirmation by histology. In other publications, the Erlangen group described the utility of virtual chromoendoscopy with *i*-Scan for the real-time diagnosis of both gastric and duodenal CD [23, 24]. Using this technique, irregular thickened folds in the duodenum, reddish areas, and especially CD-typical aphthoid erosions were characterized, which were not evident on conventional HD-WLE imaging [23]. In the stomach, *i*-Scan imaging revealed erythematous CD-associated inflammatory areas and spots as well as aphthoid lesions [24]. These virtual CE findings may facilitate an earlier diagnosis of upper gastrointestinal CD and allow targeted biopsies for histological confirmation [23, 24]. Similar findings have been reported in the colon [25].

A very recent comparative study of Iacucci and co-workers proved that *i*-Scan or HD-WLE is not inferior to dye spraying chromocolonoscopy for detection of colonic neoplastic lesions during surveillance colonoscopy [26].

Regarding optical chromoendoscopy, NBI colonoscopy has been shown to be more precise in diagnosing the degree of inflammation in patients with quiescent UC, when compared with conventional HD-WLE [27]. Furthermore, Kudo and co-workers proved a more precise grading of the inflammatory activity in UC with NBI [27] compared to histopathology (as gold standard). NBI colonoscopy was demonstrated to be a useful tool for the *in vivo* detection of angiogenesis in IBD, with a significant increase in vessel density in inflamed areas, which were NBI-positive [28]. In one case report, NBI colonoscopy was used to detect a DALM (dysplasia-associated lesion or mass) in a UC patient [29]. In a first prospective study where NBI colonoscopy was compared with conventional colonoscopy for the detection of dysplasia in patients with long-standing UC, the sensitivity of the (first generation) NBI system for neoplasia detection was similar to conventional colonoscopy, although more suspicious lesions were found during NBI [30]. Matsumoto and co-workers went further and demonstrated the value of magnification imaging with NBI for neoplasia prediction in UC [31]. Another study showed that NBI required less biopsy sampling and a shorter withdrawal time in comparison to WLE in neoplasia detection in UC patients [32]. In contrast, a recent study showed that NBI did not improve the detection of neoplasia in patients with UC compared to HD-WLE. Therefore, NBI proved unsatisfactory for differentiating neoplastic from nonneoplastic mucosa [33]. In a crossover study of 29 patients with IBD, Sussman and co-workers compared WLE, dye-based CE (indigo carmine), and NBI [34]. Hereby, WLE and CE accuracy showed superiority over NBI (64% and 63% versus 42%, resp.) in inflammation and pseudopolyp histology prediction.

A recent noninferiority crossover trial utilized WLE, dye-based CE (methylene blue), and *i*-Scan imaging during surveillance colonoscopy [35]. In this paper, Iacucci and co-workers showed a benefit of applying CE imaging in the detection and characterization of sessile serrated adenomas in IBD surveillance colonoscopies (93% sensitivity and specificity).

In a randomized trial comparing CE versus NBI, there was no significant difference in the detection of colitis-associated neoplasia, although the total procedural time was on average 7 min shorter in the NBI group [36].

Data regarding virtual CE with FICE (Fujinon intelligent chromoendoscopy) technology in IBD are currently lacking.

#### 3.2.3. Limitations

Although CE is easily applicable and ready available (push of a button on the endoscopic tip or topical dye application), there are still technologic differences in the equipment standards between endoscopy centers, especially because of high acquisition cost for HD/HD + devices. Another limitation is the lack of standardized training, as well as a lack of standardized diagnostic and staging scores applicable to the different techniques.

### 3.3. Endoscopic Ultrasound

Although endoscopic ultrasound (EUS) has an established role in conventional gastrointestinal imaging, in IBD it is not widely used. EUS is capable of providing parietal (intramural) as well as transmural and extraluminal imaging, as additional data to conventional transabdominal ultrasound. During the diagnosis and staging of IBD, characterization of the intestinal layers may be important in uncertain cases to differentiate between UC and CD. Rectal ultrasound is sometimes useful for assessment of IBD severity and for perianal fistula and abscess characterization, utilizing rigid rectal ultrasound probes [37]. Modern echo-endoscopes permit deeper EUS data acquisition beyond the sigmoid colon to assess mucosal and submucosal as well as total wall thickness and locoregional lymph nodes. In a blinded study of 52 patients, EUS was able to differentiate UC from CD and wall thickness correlated well with activity and histology [38]. When mucosal-submucosal and total wall thickness and lymph node detection were combined, the sensitivity was 92.3% for the differentiation of active UC/CD. Further, there was a strong correlation of total wall thickness with histological inflammation scores.

#### 3.3.1. Limitations

EUS defines more precisely transmural pathology and is not applicable in UC. Although it has high sensitivity, EUS is rarely used in routine IBD diagnostics.

### 3.4. Capsule Endoscopy

For diagnosing indeterminate IBD cases, for description of location and extent of inflammation in CD, and finally for therapeutic monitoring, capsule endoscopy has proved feasible and informative, especially in cases where bidirectional conventional endoscopy and enteroscopy were inconclusive or not (easily) applicable (like in paediatric adverse events [39]). Before capsule usage, radiologic exclusion of a significant bowel stenosis is mandatory (risk of hang-up and subsequent need for surgery). The first capsule endoscopy experience in IBD was published in 2004 [40]. In a large single-center study [41], capsule endoscopy findings led to changes in the management of the majority of IBD patients. Hereby, capsule endoscopy findings in 128 investigations over 6 years consisted of aphthae/ulcers (22.1%), stenosis (8.1%), and stenosis with capsule retention (17.4%). 61.6% of CD patients had a subsequent change in medication within 3 months after capsule endoscopy, as 39.5% initiated new IBD medication. Following capsule endoscopy, 12.8% of CD patients needed surgery within 3 months. Severe findings on capsule endoscopy in CD patients, as compared to no/minimal findings, resulted in significant differences in medication changes (73.2% versus 51.1%, *P* = 0.04), addition of medications (58.5% versus 22.2%, *P* < 0.01), and surgeries (21.9% versus 4.4%, *P* = 0.01) [41]. Multiple comparative studies proved the diagnostic yield superiority of 83–100% [42–44] for capsule endoscopy related to computer and magnetic resonance tomography, push enteroscopy, and even ileocolonoscopy [45].

#### 3.4.1. Limitations

The limitation of capsule endoscopy rely in its usefulness strictly in isolated cases of uncertain IBD and small bowel CD without ileus or radiologically evidenced stenosis/strictures or intestinal passage disruptions.

## 4. Advanced Endoscopic Imaging Techniques

### 4.1. Full-Spectrum Endoscopy (FUSE®)

Full-spectrum endoscopy (FUSE) is a new high-definition endoscope that incorporates supplementary lateral camera lenses (to the right and left sides of the colonoscope tip) in addition to the standard forward-viewing camera. These 3 lenses deliver a 330° panoramic field of view of the mucosa as opposed to the 170° field of view from a conventional forward-viewing colonoscope. One study demonstrated an improved visualization of the side walls, blind spots, and behind folds. The FUSE system provided a significantly decreased adenoma miss rate from 41% using forward-viewing colonoscopes (20 adenomas missed a total of 49) to 7% using FUSE (5 adenomas missed of a total of 67) in a tandem back-to-back colonoscopy study of a non-IBD population [46]. In a recent prospective study of IBD patients, panoramic imaging obtained by FUSE increased the number of dysplastic lesions detected, compared with conventional forward-viewing colonoscopy. Hereby, forward-viewing colonoscopy missed 71.4% of dysplastic lesions per lesion whereas FUSE missed 25.0% per lesion [47]. Still, further multicenter studies are necessary to confirm these data.

### 4.2. Endocytoscopy

Endocytoscopy (Olympus, Japan) is based on the optical principle of contact light microscopy, delivering *in vivo* real-time ultra-magnifying microscopic imaging of the mucosal surface at a magnification up to 1390-fold [48, 49]. Regarding the diagnostic yield of endocytoscopy in UC, Bessho et al. [50] showed in a cohort of 55 UC patients a correlation of rho = 0.713 (*P* < 0.001) between endoscopy and histopathological activity and a *κ* value of 0.79 in the validation of the proposed endocytoscopy system score (ECSS) [50]. Another study regarding the value of endocytoscopy for describing inflammatory activity in IBD showed a precise discrimination of single mucosal inflammatory cells and also the degree of inflammation [51]. The sensitivities and specificities for cytologic detection were neutrophilic (60% and 95%), basophilic (74.43% and 94.44%), and eosinophilic granulocytes (75% and 90.48%) and lymphocytes (88.89% and 93.33%), while interobserver and intraobserver agreements were 0.61–0.78 and 0.76–0.88, respectively. Concordance between EC and histopathology for grading of the intestinal disease activity was 100% [51].

Further prospective studies are needed to extend the diagnostic possibilities of this method.

### 4.3. Fluorescence and Autofluorescence Endoscopy

Both fluorescence and autofluorescence endoscopy are emerging imaging techniques that rely on visualization of fluorescence light (wavelength: 500–630 nm) emitted by either administered or endogenous fluorophores. In a prospective study of 43 patients with UC, the yield of autofluorescence imaging was superior to WLE in inflammation detection (85% versus 79%) [52]. Fluorescence endoscopy with 5-aminolevulinic acid (5-ALA) in comparison with WLE showed no significant difference in the dysplasia detection rate in IBD [53]. Finally, a crossover trimodal study comparing autofluorescence imaging with NBI and WLE showed a superiority of autofluorescence in neoplasia detection in UC [54]. Autofluorescence endoscopic studies on IBD and publications are rare, which still leave broad possibilities for further research endeavours.

### 4.4. Confocal Laser Endomicroscopy in IBD

#### 4.4.1. CLE for Assessment and Characterization of Inflammation and Mucosal Healing Prediction

**Figure 2:**
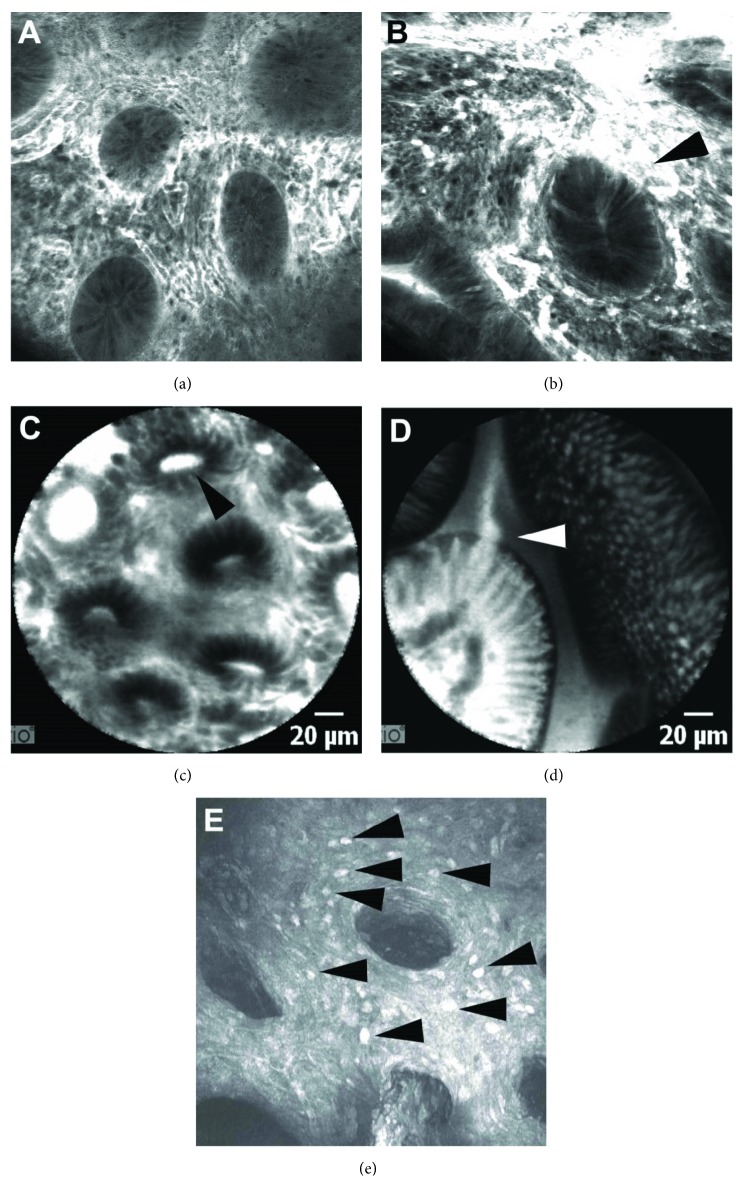
Endomicroscopic imaging in Crohn's disease and ulcerative colitis. Images (a) and (b) show endomicroscopic images provided by the integrated system (eCLE) with typical inflamed crypts and hypervascularization in Crohn's colitis (a) and ulcerative colitis (b); arrowhead points towards vascular leakage. Pictograms (c) and (d) show endomicroscopic images provided by the probe-based system (pCLE) in a Crohn's disease patient with colonic and ileal involvement (arrowhead in (c) shows a deformed crypt with lumen leakage; arrowhead in (d) shows a typical epithelial gap). (e) shows a premier molecular endomicroscopic imaging of golimumab FITC (ex vivo eCLE) in an ulcerative colitis patient that underwent proctocolectomy (arrowhead shows golimumab FITC-positive cells in the lamina propria, suggesting the high number and density of effector cells of inflammation).

Confocal laser endomicroscopy (CLE) is a sophisticated endoscopic imaging technique introduced in 2004 that allows a so-called optical biopsy. Two CLE systems are available, the integrated endoscopic system (eCLE, from PENTAX, Tokyo, Japan; Figures 2(a) and 2(b)) and the probe-based system (pCLE, from Mauna Kea Technologies, Paris, France; Figures 2(c) and 2(d)), the latter being passable through the working channel of standard endoscopes. By enabling real-time *in vivo* visualization of a plethora of novel cellular and subcellular details, which correlates with conventional histology, CLE has the potential to have a major impact on endoscopic diagnosis [49, 55, 56]. Due to its resolution and tissue penetration of approximately 250 *μ*m, endomicroscopy can describe several aspects of mucosal architecture like crypt alteration (form, density, integrity, crypt lumen distortion, crypt leakage, and goblet cell density within the crypts) as well as microvascular changes (increased vascularity, vascular integrity, and leakage). Based on these two important criteria, Li and co-workers described the first CLE classification of inflammation activity for UC [57]. Prior to this publication, in 2008, Watanabe and co-workers described the microscale mucosal changes provided by CLE between inflamed and noninflamed colon in UC [58].

The Erlangen group provided the endomicroscopic inflammation criteria for a Crohn's colitis activity score [59]. Furthermore, the gastric and duodenal manifestations of CD were described and diagnosed *in vivo*, based on high-definition and virtual chromoendoscopy-guided endomicroscopy (eCLE) [24, 60, 61]. The same group provided *in vivo* differentiation criteria between CD and UC using CLE [62]. In a prospective study, Kiesslich and co-workers [63] published data on the utility of eCLE in predicting an IBD relapse, by describing the process of cell shedding and quantifying the local epithelia barrier dysfunction. In IBD patients in clinical remission, the increase in cell shedding with fluorescein leakage was associated with subsequent relapse within 12 months [63]. Further recent advances in IBD diagnosis and outcome assessment have addressed the capacity of eCLE to evaluate and define more precisely the process of mucosal healing during standard therapy (anti-TNF antibodies) [64]. The Erlangen group prospectively validated the first CLE mucosal healing score for colonic Crohn's colitis und UC, with high sensitivity and specificity values compared with histology (Gupta Index). This score, designed for everyday use in clinical practice, used eCLE to predict mucosal healing and therapeutic outcome over a period of 3 years in UC [64]. IBD patients showing an eMHs score < 1 had a long-lasting clinical remission and reduced hospitalization, steroid, and surgery need, which qualifies the endomicroscopic mucosal healing score and the CLE implicitly as a valuable tool for prediction of a deep lasting remission [64].

Another prospective study of CLE focused on developing an intestinal permeability score in patients with IBD. An impaired intestinal permeability correlated with ongoing bowel symptoms, while an increase in permeability correlated with increased severity of diarrhea [65].

The value of CLE in IBD has been recently addressed in two Danish studies. Karstensen et al. showed that eCLE can predict a relapse in quiescent CD by highlighting fluorescence leakage and microerosions as risk factors for an inflammatory fallback in 50 IBD patients (*P* = 0.043 and *P* = 0.034, respectively; inter- and intraobserver reproducibility *κ* > 0.80 and *κ* > 0.60, meaning a good agreement) [66]. The same group described pCLE to assess the longitudinal histologic changes upon various immunosuppressive therapies in UC patients [67]. Prediction of UC relapse by pCLE, based on crypt structural and microvascular criteria, was confirmed by an Italian group [68].

#### 4.4.2. CLE in Neoplasia Detection and Surveillance in IBD

Regarding the value of CLE for detection of dysplasia-associated lesions or mass (DALM) or adenoma-like mass (ALM) in UC patients, Hurlstone and co-workers obtained high accuracy values, for example, the agreement between CLE and histopathologic evaluation was *κ* = 0.91, and accuracy was 97% [69]. Indigo carmine-aided pCLE accurately detected dysplasia in long-standing UC [70]. This study emphasized the advantages of combining imaging techniques to improve diagnostic accuracy. By using bimodal imaging, Kiesslich and co-workers showed that targeted eCLE using chromoendoscopy guidance (with methylene blue) had a significantly higher diagnostic yield (4.75-fold) for neoplasia detection in UC patients, than conventional colonoscopy with random biopsies [71]. The same work showed that this dual imaging technique reduced the need for biopsies by 50 percent. On the same issue of targeted versus random biopsies for neoplasia detection in IBD, Günther et al. [72] compared random biopsy during WLE with CE (indigo carmine and quadrantic biopsies) and eCLE (with targeted versus random biopsies). CE- and eCLE-guided targeted biopsies were more accurate in neoplasia detection in UC than random biopsies during WLE.

A similar randomized trial with methylene blue CE-guided eCLE versus WLE with random biopsies in neoplasia detection in 162 UC patients with high neoplastic risk (as IN, intraepithelial neoplasia history, or PSC) revealed no significant difference in the detection for IN (8 versus 7 patients) [73], although the targeted approach did reduce biopsy sampling, in line with Kiesslich et al.'s study [71].

Comparative studies between colonoscopy with NBI and pCLE regarding detection yield for IN have also been addressed [74]. In one study, NBI was superior to pCLE in neoplastic discrimination of sessile and pedunculated polyps in UC (sensitivity, specificity, and accuracy of 100%, 89%, and 92% versus 65%, 82%, and 81%, resp.), although different approaches were applied (blind pCLE versus real-time NBI assessment), making objective comparisons difficult.

CD surveillance studies are rarer compared to UC trials. A recent prospective study from 2016 showed a limited practical applicability for neoplasia detection in CD by CE-guided eCLE [75]. CE-guided eCLE presented a low dysplasia detection rate of IN (9.8%). The combination of CE and eCLE for differentiating neoplastic from nonneoplastic lesions had an accuracy of 86.7%, sensitivity of 42.9%, and specificity of 92.4%. For CE alone, this was 80.3% (95% CI, 70.7–89.9), 28.6% (95% CI, 5.1–69.7), and 86.4% (95% CI, 80.9–97.6).

There are only a few studies addressing molecular imaging in IBD using the CLE technique (Figure 2(e)). In a prospective study of 25 patients with CD, the Erlangen group described the feasibility of topically administered fluorescein isothiocyanate- (FITC-) conjugated adalimumab to detect intestinal membrane-bound tumour necrosis factor- (mTNF-) positive immune cells. Patients with high numbers of mTNF(+) cells had significantly higher short-term response rates to anti-TNF therapy, which was sustained over a 1-year follow-up period [76].

Although the CLE enthusiasm of the pioneering years decreased, confocal technology remains a promising tool for real-time cellular diagnosis, mucosal healing prediction [64], and molecular imaging, but reforms regarding reimbursements as well as lower acquisition cost have to promote a broader implementation of CLE from study settings into real-life daily routine.

#### 4.4.3. CLE Limitations

Despite the potential of this technique, CLE has limitations including costs issues (inadequate reimbursement in Europe, expensive acquisition) and limited accessibility since it is available only in large, mostly academic endoscopy centers. One of the apparent limitations of the eCLE both in multicenter studies [75] and single-center experience [50] were dysfunctions of the laser unit (of the eCLE system). Another limitation in most CLE studies, with pCLE and eCLE, is an unavoidable bias in the collection of CLE data, influenced by the surface pattern seen on conventional endoscopy. This limitation is unavoidable, and a blinded CLE sampling is impossible because the CLE technique in itself represents a point technique that needs direct and targeted contact with the mucosal area of interest. Here, the CLE sampling has to be done in areas of the colorectum with representative findings (e.g., inflammation), since a complete CLE investigation of the entire colon and rectum is impossible. In this regard, the general limitation of sampling error has to be underlined, since the technique allows only the analysis of a very small mucosal area compared to the real surface extent of diseases like IBD. Further, CLE imaging as supplementary acquisition method requires extra time, which logically extends the entire investigation time and needs higher sedation need.

## 5. Conclusions and Perspectives

An increased number of diagnostic and surveillance imaging novelties like multiband confocal imaging or multimodal studies that combine more than two advanced endoscopic imaging techniques will most probably bring in the future new insights for better diagnosis and management of the two IBD entities, CD and UC. Before more widespread adoption of these techniques, specific limitations of the describe techniques must also be addressed, including limited availability because of high acquisition costs and insufficient reimbursement, lack of standardized training and diagnostic scores, and the additional time necessary for investigation. Better reimbursement rates are needed in order to translate techniques like CLE from the experimental levels into the wide daily practice.

In the near future, techniques like the dual-band or multiband endomicroscopy will enrich the endoscopic armamentarium, allowing the usage of 2 fluorophores and more complex molecular imaging. Further, technical developments with slimmer and more flexible endoscopes, as well as improvements in digital optics like three-dimensional endoscopy and ultra-high-definition imaging (UHD/4K), are expected to enter into endoscopic production.

In conclusion, a targeted approach combining several imaging technologies in IBD diagnosis brings advantages regarding accuracy and reduces the necessity of classical forceps biopsy, as well as overall risks, which should encourage the adoption, implementation, and standardization of these modern techniques into clinical practice.
